# Utility and limitations of *Spa*-typing in understanding the epidemiology of *staphylococcus aureus* bacteraemia isolates in a single University Hospital

**DOI:** 10.1186/1756-0500-6-398

**Published:** 2013-10-02

**Authors:** Giovanni Satta, Clare Louise Ling, Emma Shelley Cunningham, Timothy Daniel McHugh, Susan Hopkins

**Affiliations:** 1 Department of Medical Microbiology, Royal Free London NHS Foundation Trust, London, UK; 2 Health Protection Agency, London, UK; 3 Division of Infection and Immunity, Centre for Clinical Microbiology, University College London, London, UK

**Keywords:** *Staphylococcus aureus*, *Spa*-typing, Epidemiology

## Abstract

**Background:**

*Staphylococcus aureus* (SA) is a recognized cause of nosocomial infections with 8,767 SA bacteraemia (SAB) cases reported in England only in 2012. Different typing methods have been developed but they are not generally performed as a routine investigation in hospital laboratories.

**Findings:**

We collected epidemiological data and *spa*-typed all SAB isolates over a 12 months period. *Spa*-typing was useful to detect two potential outbreaks of methicillin-sensitive SA (MSSA). In addition, the analysis of *spa*-types from individuals with multiple bacteriaemias helped to distinguish between relapse and re-infection.

**Conclusions:**

*Spa*-typing could be used as a rapid tool to understand the epidemiology of SAB, in particular the detection of hospital clusters and to distinguish relapse from re-infection, but clinicians should be aware of its possible limitations.

## Short report

*Staphylococcus aureus* (SA) is a leading cause of human disease and nosocomial infections. In England only, 8,767 episodes of SA bacteraemia (SAB) were reported in 2012 [1]. Mortality rate is around 30% and 50% of patients will experience a complicated infection [2].

Since 2007, England has seen a rapid decline in MRSA bacteraemias by more than 75% which has not been matched by an equivalent decline in MSSA bacteraemias [1]. Data suggests that two thirds of UK MSSA bacteraemias are healthcare associated, predominantly relating to central venous catheters or surgical site infection [3]. Thus, greater understanding of the molecular epidemiology of all SAB is an invaluable infection control tool.

*Spa*-typing, a typing method based on the DNA sequence analysis of the protein A gene, has been adopted as standard typing method by reference laboratories in several European countries [4,5] and previous studies demonstrated a good correlation between the clonal groupings obtained by *spa*-typing and those obtained by other typing techniques [6].

The primary hypothesis of this study was to test whether any outbreaks had occurred in the clinical areas with high rates of SAB. The secondary hypothesis was to determine whether patients with multiple SABs (>30 days interval with a negative blood culture between episodes) were relapses or re-infections.

We prospectively collected 82 SA isolates with associated clinical data from bacteraemias over a 12 month period from the Royal Free London NHS Foundation Trust. *Spa*-typing was performed as previously described [7] and converted to a real-time PCR platform.

A healthcare associated (HCA) SAB was defined as any SA bacteraemia positive 48 hours after admission, OR related to a device or procedure (central venous catheter, urinary catheter, surgery, etc.) OR in a patient that was a regular day attender or in-patient in previous 30 days. A cluster was defined as two or more cases geo-temporally related, where same ward/clinical team was considered as geographically related and within 30 days as temporally related. Multiple SABs from same patient were considered only in case of more than 30 days interval between episodes and with a negative blood culture during this interval.

Cases without an identifiable source of infection, with no other positive microbiology results and other negative investigations (i.e. negative echocardiogram, bone scan and other imaging) were considered as unknown source.

75 individuals accounted for 82 separate SAB. 5 individuals had 2 SAB and 1 individual had 3 SAB events. In total, 84% (n = 69) were MSSA and 16%(n = 13) were MRSA. The average age was 54 (range 3–90 years old) with a male–female ratio of 68%-32% respectively (Table 1).

**Table 1:** **Epidemiology of *Staphylococcus aureus *bacteriaemias**

**Healthcare associated (HCA) vs non HCA**	**HCA**	**Not HCA**	**Total**
	**65% (n=53)**	**35% (n=29)**	**100% (n=82)**
**Age (mean)**	55 (3–90)	51 (16–90)	54 (3–90)
**% Male–female**	M 64% - F 36%	M 76% - F 24%	M 68% - F 32%
**MSSA vs MRSA**	83% (44) -17% (9)	86% (25) -14% (4)	84% (69) -16% (13)
**30days mortality**	21% (11)	17% (5)	20% (16)
**Source:**			
**- Line related**	25 (47%)	0	25 (30%)
**- Post-op infection**	14 (26%)	0	14 (17%)
**- Urinary catheter**	1 (2%)	0	1 (1%)
**- Endocarditis**	1 (2%)	6 (21%)	7 (9%)
**- Cellulitis/soft tissue infection**	0	9 (31%)	9 (11%)
**- Ostemyelitis/joint infection**	2 (4%)	7 (24%)	9 (11%)
**- Pneumonia**	1 (2%)	2 (7%)	3 (4%)
**- Other**	0	2 (7%)	2 (2%)
**- Unknown**	9 (17%)	3 (10%)	12 (15%)
**Total (n)**	53 (100%)	29 (100%)	82 (100%)
**Specialty:**			
**- Renal**	27 (51%)	3 (10%)	30 (37%)
**- Haematology/oncology**	4 (7%)	6 (21%)	10 (12%)
**- Infectious diseases**	1 (2%)	7 (25%)	8 (10%)
**- Hepatology**	5 (9%)	2 (7%)	7 (9%)
**- Geriatrics/general medicine**	4 (8%)	2 (7%)	6 (7%)
**- Surgical group**	3 (6%)	3 (10%)	6 (7%)
**- Emergency (community)**	0	5 (17%)	5 (6%)
**- Gynaecology**	4 (7%)	0	4 (5%)
**- Intensive Care Unit**	3 (6%)	0	3 (4%)
**- Paediatrics**	1 (2%)	1 (3%)	2 (2%)
**- Other**	1 (2%)	0	1 (1%)
**Total (n)**	53 (100%)	29 (100%)	82 (100%)
**List of 45 identified *spa*-types and percentages:**			
**Most frequent**	**-***t002* (6% - n=7)		
	**-***t008*, *t127*, *t318* (all 3% - n=4)		
	**-***t021* (2% - n=3)		
**Other *spa*-types**	**-***t012*, *t018*, *t062*, *t084*, *t160*, *t701*, *t304*, *t1499*, *t1778*, *t5475* (all n=2)		
	**-***t015*, *t020*, *t032*, *t037*, *t105*, *t122*, *t164*, *t177*, *t223*, *t267*, *t346*, *t399*, *t571*, *t601*, *t665*, *t706*, *t878*, *t910*, *t937*, *t1245*, *t1467*, *t2365*, *t2956*, *t3225*, *t3732*, *t3896*, *t5078*, *t5848*, *t6265*, *t6362*, *t6509* (all n=1)		
	**-***Spa*-types not in the database (n=7)^1^		
	**-** No PCR product (n=2)^2^		

^1^Unknown *spa*-types can be send to Ridom Server for *spa*-type designation (www.spaserver.ridom.de).

^2^This is rare event with *spa*-typing but an alternative forward primer is available [6].

Using the pre-determined definitions, 65%(n = 53) of SABs were considered as healthcare associated (HCA) and 35% not HCA. There were no significant differences between the two groups (HCA versus not HCA) in terms of MSSA/MRSA ratio, age, sex (all p-values > or = 0.2, Pearson chi square test). The overall mortality rate at 30 days of collection of the positive blood culture was 20% (n = 16) and there was no significant difference between the HCA and non HCA groups (p-value = 0.7). Considering the source of infection, 47% of the total number of SABs was line and post-operative related but these sources counted up to 73% of the HCA bacteriaemias. On the other hand, common sources of non HCA were skin, soft tissue, bone and joint infections (22% of the total but 55% of the not HCA). In the 15% of cases it was not possible to identify a source of infection (17% and 10% in the HCA and non HCA respectively) (Table 1). Renal and haematology wards/clinical teams had the highest number of cases and counted for almost half (49%) of the total, followed by infectious diseases, hepatology, general medicine and surgery (Table 1).

We identified 45 different *spa* types, with type *t002* as the most frequent (6% of isolates - n = 7), followed by types *t008*, *t127* and *t318* (3% each - n = 4) (Table 1). There was a uniform distribution of all different *spa*-types within the HCA and non HCA groups and an equal variety of *spa*-types when comparing MSSA versus MRSA. Two clusters were possibly identified in the Renal and Haematology Units (all MSSA): 4 patients from the renal dialysis unit developed SAB (all *spa* type *t002*) within a 30 days period and 2 haematology patients on the same ward developed SAB within the same week (both *spa* type *t1778*).

Considering the isolates from individuals with multiple bacteraemias, 3 patients had the same *spa*-type identified, 2 patients (KH and AM) had a different *spa*-type and the patient (HT) with 3 SAB episodes had the same *spa*-type for the first two episodes but a different one in the third episode. For the three patients (KH, AM and HT) with a query re-infection, the analysis of *spa*-repeats showed these cases were caused by different strains. KH had *spa*-type *t084* (spa-repeats: 07-23-12-34-38-12-12-23-182-12-23) on the first episode and *spa*-type *t1499* (spa-repeats: 26-23-16-23-31-29-17-31-29-17-25-17-25-16-28) on the second one. AM had *spa*-type *t002* (spa-repeats: 26-23-17-34-17-20-17-12-17-16) on the first episode and *spa*-type *t021* (spa-repeats: 15-12-16-02-16-02-25-17-24) on the second one. HT had *spa*-type *t318* (spa-repeats: 15-12-16-16-02-16-02) on the first and second episodes and *spa*-type *t164* (spa-repeats: 07-06-17-21-34-34-22-34) on the third one.

Several typing systems have been developed: pulsed-field gel electrophoresis (PFGE), multilocus enzyme electrophoresis (MLEE), *spa*-typing and multilocus sequence typing (MLST). Sequence-based typing methods, such as *spa*-typing and MLST, have the advantages to be easy to perform, reproducible and comparable rather than other band-based typing methods (i.e., PFGE and MLEE) [8]. Nevertheless, *spa*-typing has also certain limitations. The major drawback of this method based on a single-locus typing is that it can misclassify particular types due to recombination and/or sequencing errors [9]. The use of *spa*-typing detected two potential clusters of MSSA within the Renal and the Haematology units. The role of spa-typing in outbreak investigations still need to be completely evaluated and it may be limited due to its low discriminatory power in comparison to other gold standard methods such as PFGE [9]. It is known that the same *spa-*types may be distributed widely amongst many unrelated PFGE patters within an epidemic group and in some cases even amongst different MLST types. Also, *spa*-type *t002* has been confirmed to be the most frequent among MSSA in a study involving 26 European countries [10]. For all these reasons, It would be valuable to confirm the presence of an outbreak with a second alternative typing method (traditionally PFGE) and a recent paper even advised the role of whole genome sequencing (WGS) in tracing person-to-person transmission and confirming hospital outbreaks [11]. However, the lack of WGS facilities and the need of a bioinformatics expertise may represent as the major limitations in the routine practice.

The identification of the same *spa*-types from individuals with recurrent bacteraemias suggested that half of these cases were relapses of the original infection. In all of them, the source of infection was not surgically removed and a medical management was tried in first instance (prosthetic joint infection and endocarditis). The *spa*-typing of the other cases with multiple bacteriaemias suggested these cases were adequately treated at the time of first episode and subsequent episodes were re-infections rather than relapses. This is useful in determining the course of treatment and investigation of these patients, but also suggests innate individual risk factors for SAB. The analysis of *spa*-repeats showed these cases were caused by different strains. Recently, the implementation of the based upon repeat patterns (BURP) algorithm to the StaphType software has greatly facilitated the assignment of *spa*-types into clonal complexes and singletons, partially overcoming the risk of SNPs and sequencing errors [12], but this remains another possible limitation to remember.

In conclusion, we used *spa*-typing as a rapid typing method to identify hospital clusters and to distinguish between SAB relapse and re-infection. It can be a useful and cost-effective routine screening tool in a hospital setting with high incidence of SAB, but clinicians should be aware of its limitations and the need to confirm hospital outbreaks with a second typing method.

## Ethical approval

No Ethical Approval was needed for this project as the isolates used were collected as part of routine clinical service. However, a DRAG (Departmental Research Advisory Group) approval was required.

## Competing interests

The authors declare that they have no competing interests.

## Authors’ contributions

GS and SH conceived of the study. CL designed the molecular work and helped with the alignment. GS carried out the molecular work, data collection and drafted the manuscript. ESC participated in the molecular work. TDM and SH helped to draft the manuscript and helped with coordination of the work. All authors contributed and approved the final manuscript.
